# A prospective study of hearing changes after beginning zidovudine or didanosine in HIV-1 treatment-naïve people

**DOI:** 10.1186/1471-2334-6-28

**Published:** 2006-02-20

**Authors:** Jeffrey T Schouten, David W Lockhart, Thomas S Rees, Ann C Collier, Christina M Marra

**Affiliations:** 1 Department of Surgery, University of Washington, Seattle, USA; 2 Department of Biostatistics and Center for AIDS Research, University of Washington, Seattle, USA; 3 Department of Otolaryngology- Head and Neck Surgery, University of Washington, Seattle, USA; 4 Deparment of Medicine, Allergy and Infectious Diseases, University of Washington, Seattle, USA; 5 Department of Neurology, University of Washington, Seattle, USA

## Abstract

**Background:**

While hearing loss in HIV-infected people after beginning nucleoside reverse transcriptase inhibitors (NRTIs) has been reported, there have been no prospective studies that measured hearing changes longitudinally in treatment-naïve HIV-infected subjects following initiation of regimens containing NRTIs. The goal of this study was to conduct a prospective assessment of the contribution of zidovudine (ZDV) and didanosine (ddI) to hearing loss

**Methods/design:**

A prospective observational pilot study to determine whether ZDV or ddI, alone or in combination, are associated with sensorineural hearing loss in HIV-infected persons. Changes in hearing levels at all frequencies and in low and high frequency pure tone averages were measured at baseline, 16, and 32 weeks after initiating antiretroviral therapy.

**Discussion:**

Treatment with ZDV and ddI did not result in loss of hearing, even after taking into account noise exposure, immune status and age. The results of this prospective pilot study do not support the notion that treatment with nucleoside antiretrovirals damages hearing.

## Background

Cross-sectional studies and case reports show that hearing loss may be common among HIV-infected people [1-3]. Hearing loss may be associated with HIV infection itself, opportunistic infections, or ototoxic drug therapy [1]. However, in up to 50% of HIV-infected people with hearing loss, no cause can be identified [4]. In a prior cross sectional study, we showed that hearing loss was common in HIV-infected people and was associated with older age and antiretroviral use, but we were unable to determine an association with specific antiretroviral agents [1].

While hearing loss in HIV-infected people after beginning nucleoside reverse transcriptase inhibitors (NRTIs) has been reported [5], there have been no prospective studies that measured hearing changes longitudinally in treatment-naïve HIV-infected subjects following initiation of regimens containing NRTIs. The goal of this study was to conduct a prospective assessment of the contribution of zidovudine (ZDV) and didanosine (ddI) to hearing loss.

## Methods/design

### Study design

Thirty three antiretroviral-naïve subjects with their most recent peripheral blood CD4+ T-cells > 200/ul performed within the past 3 months were prospectively enrolled in this study between January 1996 and December 1999. Subjects were recruited from one research and one outpatient medical clinic for persons with HIV-1. (An AIDS Clinical Trials Unit and a public, hospital-based, HIV primary care clinic staffed by university faculty and ID fellows.) All subjects initiated therapy with regimens containing either ZDV or ddI, and although use of only one of these agents was preferred, use of both was allowed. People with prior hearing loss requiring hearing aids, and those with active substance abuse that would interfere with their participation in the trial were excluded. All subjects signed an Institutional Review Board-approved consent prior to participation in this study.

Subjects underwent audiometry prior to initiating antiretroviral therapy (entry) and at weeks 16 and 32. At each visit, plasma HIV RNA and CD4+ T-cells were measured.

### Audiometry

All audiometric testing was completed by a certified audiologist or under their direct supervision. Following otoscopic inspection, tympanometry screening was performed to rule out significant middle ear pathology. Audiometric testing was completed with the use of a clinical diagnostic audiometer (Grason Stadler, Model GSI 61) in a sound-treated test booth. Air conduction thresholds were obtained for each ear at 250, 500, 1000, 2000, 3000, 4000, 6000, 8000 and 12000 hertz (Hz). Pure tone bone conduction testing was administered on those persons demonstrating hearing loss by air conduction. Hearing levels were measured at 250, 500, 1000, 2000, 3000, 4000, 6000, 8000 and 12000 Hz by formal audiometry. "Hearing Level" was defined as the intensity of the sound (in dB – decibels) needed to reach threshold (both for air conduction and bone conduction). Normal hearing is generally considered to be 0–25 dB hearing level [6]. Pure tone hearing sensitivity was tested using air conduction (with earphones, which tests the entire auditory system), and then by bone conduction (using an oscillator behind the ear, which bypasses the outer and middle ear). The comparison of air conduction thresholds and bone conduction thresholds can differentiate a sensorineural hearing loss versus a conductive hearing loss.

### HIV-1 RNA assays

Plasma HIV-1 RNA was measured using the Roche UltraSensitive PCR Amplicor HIV-1 Monitor test (Roche Molecular Diagnostics, Branchburg, New Jersey). Values below the limit of detection (50 c/ml) were imputed to be 25. All viral loads were log_10 _transformed for analysis.

### Data analysis

Due to the lack of a standard definition of ototoxicity, we evaluated changes in hearing using several methods [7]. Associations between categorical and continuous variables were assessed by Chi-square or t-tests. The association between baseline hearing level and baseline health measures was examined using linear regression with jackknifed robust standard errors to protect estimation against violation of the equivariance assumption [8]. Hearing levels at 16 and 32 weeks were analyzed as the change from baseline using generalized estimating equations (GEE) with an exchangeable working correlation matrix, an identity link function and Gaussian error distribution. The GEE approach was chosen to allow use of data from both visits while appropriately accounting for the correlation between the 16 week and 32 week measurements of the same patient [9]. In the GEE models, patients were classified as on ZDV (or ddI) if they had taken it at any prior time in the study. Two patients began ZDV, and one patient began ddI, between 16 and 32 weeks. All three were classified in the no drug group at 16 weeks and the drug group at 32 weeks. Hearing level in the left and right ears was averaged to generate a single value at each frequency for each individual. In addition to the individual frequencies, a low frequency pure tone average (PTA) was computed as the mean in both ears at 500, 1000 and 2000 Hz and a high frequency PTA was computed as the mean in both ears at 4000, 8000 and 12000 Hz. Missing individual frequency hearing levels occurred in 11 subjects at one of the three observations and these values were imputed for the purposes of the analysis. These values were calculated based on the average of the adjacent higher and lower frequency hearing level measured at that visit. P values of ≤ 0.05 were considered significant, and the median age of 35 years was chosen to dichotomize age in the analysis of age effects.

## Discussion

### Findings at the entry visit

The demographics of the 33 subjects included in the analysis, shown in Table 1, were representative of the epidemic in the local area at the time this study was accrued: primarily gay, white men. Subjects were well balanced with regard to baseline hearing in the ZDV versus ddI exposure group in terms of their demographic characteristics. However, the subjects who began taking ddI were healthier at study entry than those who initiated therapy with ZDV, with lower plasma HIV RNA levels and significantly higher CD4+ T-cell counts.

**Table 1:** Patient demographic characteristics at entry. (P-values are from t-tests comparing those who took each drug to those who did not.)

	All Subjects	Subjects with follow up	Exposed to ZDV*	Exposed to ddI†
N	33	23	19	7
Age in years: Mean (Range)	35.3 (23 – 51)	35.5 (23 – 51)	36.2 (23 – 51) (p = .27)	35.4 (25 – 51) (p = .97)
Male, N (%)	31 (94%)	21 (91%)	18 (95%) (p = .20)	6 (86%) (p = .53)
Race, N (%)				
White Black Hispanic Asian	24 (73%) 3 (9%) 1 (3%) 5 (15%)	16(70%) 2 (9%) 1 (4%) 4 (17%)	14 (74%) 1 (5%) 0 (0%) 4 (21%) (p = .062)	6 (86%) 0 (0%) 1 (14%) 0 (0%) (p = .15)
CD4+ T-cells/ul Mean (Range)	483 (213 – 1130)	481 (213 – 960)	472 (213 – 960) (p = .64)	714 (444 – 960) (p < .0001)
Plasma HIV RNA‡ 1000 copies per/ml Mean (Range)	102 (0.1 – 670) (N = 31)	75 (0.1 – 505) (N = 22)	87 (0.1 – 505) (p = .69)	2.8 (0.1 – 8.0) (p = .0006)
Low Frequency PTA§in dB Mean (Range)	11 (0 – 40) (N = 32)	12 (0 – 40)	14 (1 – 40) (p = .41)	16 (0.5 – 40) (p = .40)
High Frequency PTA in dB Mean (Range)	20 (0.8 – 51) (N = 32)	22 (1.7 – 51)	22 (2 – 51) (p = .98)	23 (3 – 50) (p = .64)

*ZDV = zidovudine, †ddI = didanosine, ‡RNA = ribonucleic acid, §PTA = pure tone average

At study entry, 22 of 33 subjects had a hearing level greater than 25 dB at one or more frequencies in one or both ears, 16 had at least one hearing level greater than 40 dB and 7 had at least one hearing level greater than 60 dB. Most of the decreased hearing sensitivity was at higher frequencies. No one had a hearing level above 40 dB in both ears at 2000 Hz or lower, whereas 9 subjects had a hearing level above 40 dB in both ears at 12000 Hz, 2 subjects had a hearing level above 40 dB in both ears at 6000 and 8000 Hz, and 3 subjects had a hearing level above 40 dB in both ears at 3000 and 4000 Hz. Only 4 subjects reported a history of occupational sound exposure, and 12 subjects reported a history of recreational sound exposure. Tinnitus was reported at entry in 14/33 (42%) subjects.

Low and high frequency PTA at baseline were not significantly associated with baseline viral load or baseline CD4+ T-cell count. (Table 2)

**Table 2:** Mean difference in hearing level (HL) at baseline and mean change in hearing level associated with variation in baseline plasma HIV RNA and baseline CD4+ T-cell count.*

	Baseline Plasma HIV RNA (Population difference in dB per 10-fold *increase*)		Baseline CD4+ T-cell count (Population difference in dB per 100 cells/mL *increase*)	
	Baseline HL	Change in HL†	Baseline HL	Change in HL†

Low Frequency PTA‡	-2.3 (-7.9 – 3.2) p = 0.395	-0.016 (-0.93 – 0.90) p = 0.333	0.50 (-1.7 – 2.7) p = 0.646	- 0.27 (-0.86 – 0.31) p = 0.354
High Frequency PTA	-0.44 (-7.9 – 7.0) p = 0.906	0.34 (-1.7 – 2.4) p = 0.747	-0.29 (-2.7 – 2.2) p = 0.801	0.089 (-0.71 – 0.53) p = 0.780

*A negative value indicates improved hearing and a positive value indicates worse hearing

†Adjusted for baseline pure tone average and time since baseline.

‡PTA = Pure Tone Average

Two subjects had a history of an AIDS-defining illness (one subject with previous *Pneumocystis jiroveci *pneumonia and one with previous esophageal candidiasis). Their mean low frequency PTA was the same as the subjects without an AIDS diagnosis (11 dB). The mean high frequency PTA for subjects with an AIDS diagnosis (28 dB) was higher than subjects without an AIDS diagnosis (20 dB), but the difference was not significant (p = 0.40).

### Findings at follow-up visits

Twenty-two of the 33 subjects returned at 16 weeks and nineteen returned at 32 weeks. Four patients had a 16-week visit and no 32-week visit and one patient had a 32-week visit and no 16-week visit. Subjects with follow up had a trend toward worse hearing at baseline, compared to baseline hearing in subjects who did not follow up, but these differences were not significant. Specifically, the baseline low frequency PTA in those with follow-up was 5.1 dB higher than in those without follow-up (p = .19) and the baseline high frequency PTA in those with follow-up was 6.5 dB higher than in those without follow-up (p = .22). The difference in baseline high and low frequency PTAs between those with and without a 16-week visit is similar to the difference between those with and without a 32-week visit, and both are similar to the difference between those with any follow up and those with no follow up. Those with follow up had lower plasma HIV RNA concentration at entry (0.74 log_10 _copies/ml lower, p = 0.046), but peripheral blood CD4+ T-cell count at entry did not differ between the two groups (p = 0.94).

Although patients with higher CD4+ T-cell counts at entry had greater improvement in low frequency PTA, this difference did not reach statistical significance (Table 2).

Table 3 shows the change in hearing level for each frequency at each time point for subjects in both treatment groups combined. Overall, there was a trend towards improvement, but it did not reach clinical (20 dB or more at 1 frequency) nor statistical significance.

**Table 3:** Mean and standard deviation of hearing level in dB for each frequency at baseline and mean and standard deviation of change in hearing level (average of R & L ear).

Frequency (Hz)	Baseline – All subjects (dB)	Baseline – Subjects with follow up (dB)	Change at 16 wks* (dB)	Change at 32 wks* (dB)
Number of subjects	33	23	22	19
250	13.3 +/- 8.0	14.8 +/- 8.3	-1.6 +/- 5.1	-2.1 +/- 5.1
500	11.3 +/- 8.6	12.7 +/- 9.8	-0.6 +/- 5.1	-2.1 +/- 5.1
1000	11.2 +/- 9.8	11.4 +/- 11.3	-0.6 +/- 3.2	0.0 +/- 3.0
2000	9.6 +/- 12.0	11.3 +/- 13.1	-0.5 +/- 3.9	0.0 +/- 2.8
3000	13.6 +/- 13.7	16.1 +/- 14.8	-0.3 +/- 4.5	0.0 +/- 2.8
4000	17.4 +/- 14.9	19.1 +/- 15.8	-0.9 +/- 4.5	-1.1 +/- 5.7
6000	20.5 +/- 13.7	22.6 +/- 14.7	0.6 +/- 5.6	-0.2 +/- 6.9
8000	23.4 +/- 14.5	25.0 +/- 15.0	-2.2 +/- 5.6	-0.4 +/- 7.2
12000	36.3 +/- 19.7	37.9 +/- 19.1	-0.6 +/- 8.4	0.0 +/- 11.4
Low Freq PTA	11.5 +/- 9.8	12.1 +/- 10.8	-0.6 +/- 2.5	-0.9 +/- 2.0
High Freq PTA	20.4 +/- 13.4	22.2 +/- 14.2	-0.8 +/- 4.8	-0.6 +/- 5.2

*A negative value indicates improved hearing and a positive value indicates worse hearing

Table 4 shows estimates and 95% confidence intervals for the change in PTA associated with taking ZDV and ddI from GEE models adjusting for week, baseline PTA and age. There were no significant changes in hearing over the 32 weeks of observation in subjects taking ZDV or ddI, even taking into account age.

**Table 4:** Mean difference in, and 95% confidence interval for, change from baseline hearing level in dB for patients receiving regimens containing ZDV or ddI, estimated by GEE model adjusting for time since beginning therapy, baseline hearing level, and age.*

	Low Frequency PTA†			High Frequency PTA		
	All patients	Age < 35 years	Age ≥ 35 years	All patients	Age < 35 years	Age ≥ 35 years

ZDV	4.0 (-1.4 – 9.5)	3.9 (-0.6 – 8.4)	4.5 (-2.1 – 11.0) p = 0.70	1.2 (-1.2 – 3.5)	1.5 (-1.7 – 4.8)	0.80 (-2.4 – 4.0) p = 0.75
ddI	- 2.6 (-6.1 – 0.9) p = 0.91	- 0.6 (-2.5 – 1.2)	- 5.4 (-13.8 – 3.1) p = 0.31	0.2 (-3.1 – 2.7)	- 0.2 (-3.3 – 2.8)	- 0.1 (-3.7 – 3.5) p = 0.95
Combined (per 100 drug-days) ‡	- 0.08 (-1.2 – 1.0 ) p = 0.89	-0.28 (-0.80 – 1.4) p = 0.61	-0.45 (-1.6 – 0.69) p = 0.44	-0.047 (-1.4 – 1.3) p = 0.75	0.098 (-2.0 – 1.3) p = 0.92	-0.22 (-1.6 – 1.2) p = 0.70

*A positive value indicates worsening hearing level; a negative value indicates improved hearing level.

†Pure Tone Average

‡Cumulative total number of days each patient took ZDV and ddI

We also used GEE models to examine the dose-response relationship of either ZDV or ddI together by adding together the number of days each patient took each drug; thus, a patient who was taking both drugs for 60 days had 120 drug-days of exposure and a patient taking ZDV but not ddI for 30 days who then switched to ddI for 30 days would have 60 drug-days of exposure. Longer treatment with ZDV and ddI did not influence change in pure tone averages.

### Influence of noise exposure and tinnitus

At baseline, the low and high frequency PTAs for those with a history of occupational or recreational noise exposure did not differ from those without such exposure (data not shown). Similarly, the change in PTAs did not differ between the two groups (data not shown). While there was a trend to decreased tinnitus reported at week 16, (7/21, 33%) and week 32 (3/19, 16%), compared to 42% at baseline, these changes did not reach statistical significance. (p = .49 at 16 weeks, and p = .053 at 32 weeks).

We conducted this longitudinal study to evaluate changes in hearing following the initiation of therapy with ZDV, ddI, or both drugs in antiretroviral therapy (ART)-naïve subjects. There were no significant changes in hearing at 16 and 32 weeks, even taking into account noise exposure, CD4+ T-cell count and plasma viral load. Our previous cross-sectional study, which was larger (99 versus 24 subjects), showed an interaction between antiretroviral use and age [1]. Limitations of our present study include small sample size, lack of randomization to ZDV or ddI, duration of follow up, and lack of a control group. The strength of our study is the use of detailed audiometry before and after beginning NRTIs in treatment-naïve subjects.

Prior cross-sectional studies and case reports have shown an association between hearing loss and NRTI therapy [1-3]. The results of the current prospective study do not confirm this relationship and are consistent with the report from the Adult/Adolescent Spectrum of HIV Disease Project Group that demonstrated no association between hearing loss and age. Of note, however, that study was based on a retrospective chart review for ICD-9 coding for hearing loss and not on formal audiometry [3].

There have been two case reports of hearing loss in subjects receiving ART regimens that included NRTIs and a second class of antiretrovirals; one with a non-nucleoside reverse transcriptase inhibitor (NNRTI) (nevirapine) and one with a protease inhibitor (PI) (lopinavir/ritonavir) each combined with NRTIs, (both these subjects were also receiving stavudine and lamivudine). One case reported sudden hearing loss two weeks after the person completed one month of post-exposure prophylaxis which resulted in long-term hearing loss [10]. The other case reported hearing loss in a subject with extensive HIV pretreatment, and suggested a possible relationship with the protease inhibitor, although the were other possible explanations noted in Simdon's reply to this case report [11,12]. Simdon reported three subjects who experienced ototoxicity, all of whom were over the age of 45 and received combination ART with 2–3 NRTIs plus a NNRTI or a PI. All 3 of the subjects had prior hearing problems, prior exposure to occupational noise and all developed significant tinnitus [2]. The authors suggested that NRTIs should be used sparingly in patients with preexisting hearing loss. While our study excluded subjects with prior hearing loss requiring hearing aids and included younger participants, several subjects had baseline hearing loss yet we saw no deterioration in hearing after beginning NRTIs. Also, we observed a trend towards less tinnitus over the 32 weeks of nucleoside therapy.

Our finding of little impact on hearing after beginning ZDV or ddI is reassuring and suggests that, if such agents do cause hearing loss, it is uncommon. Alternatively, baseline hearing impairment due to HIV itself may improve with improved immune status as a result of antiretroviral treatment, masking any potential ototoxicity. This hypothesis could be tested in a future study by comparing hearing changes in subjects who do and do not experience improved immune status after beginning NRTIs.

## Abbreviations

ART- antiretroviral therapy

dB- decibels

ddI- didanosine

GEE- generalized estimating equations

HL- hearing level

Hz- hertz

NNRTI- non-nucleoside reverse transcriptase inhibitor

NRTI- nucleoside reverse transcriptase inhibitor

PI- protease inhibitor

PTA- pure tone average

RNA- ribonucleic acid

ZDV- zidovudine

## Competing interests

The author(s) declare they have no competing interests.

## Authors' contributions

JTS, DWL, TSR, ACC, and CMM have made substantial contributions to conception and design, or acquisition of data, or analysis and interpretation of data; have been involved in drafting the manuscript or revising it critically for important intellectual content; and have given final approval of the version to be published. The principal investigator, CMM, and JTS had full access to all the data in the study and take responsibility for the integrity of the data and the accuracy of the data analysis.

The funding agencies were not in control of the design and conduct of the study; collection, management, analysis, or interpretation of the data; or preparation, review, or approval of the manuscript.

## Pre-publication history

The pre-publication history for this paper can be accessed here:


